# The lower He-sea points playing a significant role in postoperative ileus in colorectal cancer treated with acupuncture: based on machine-learning

**DOI:** 10.3389/fonc.2023.1206196

**Published:** 2023-07-24

**Authors:** Xu Zhang, Wenjing Yang, Junliang Shang, Wenchao Dan, Lin Shi, Li Tong, Guowang Yang

**Affiliations:** ^1^Department of Nutrition, Beijing Hospital of Traditional Chinese Medicine, Capital Medical University, Beijing, China; ^2^Department of Oncology, Beijing Hospital of Traditional Chinese Medicine, Capital Medical University, Beijing, China; ^3^Department of Surgery, Beijing Hospital of Traditional Chinese Medicine, Capital Medical University, Beijing, China; ^4^School of Graduates, Capital Medical University, Beijing, China

**Keywords:** acupuncture, postoperative ileus, colorectal cancer, machine-learning, association rules

## Abstract

**Background:**

Postoperative ileus (POI) is a common complication following abdominal surgery, which can lead to significant negative impacts on patients’ well-being and healthcare costs. However, the efficacy of current treatments is not satisfactory. The purpose of this study was to evaluate the therapeutic effects of acupuncture intervention and explore the regulation of acupoint selection for treating POI in colorectal cancer (CRC) patients.

**Methods:**

We searched eight electronic databases to identify randomized controlled trials (RCTs) on acupuncture for POI in CRC and conducted a meta-analysis. Subsequently, we utilized the Apriori algorithm and the Frequent pattern growth algorithm, in conjunction with complex network and cluster analysis, to identify association rules of acupoints.

**Results:**

The meta-analysis showed that acupuncture led to significant reductions in time to first defecation (MD=-20.93, 95%CI: -25.35, -16.51; *I*^2 =^ 93.0%; p < 0.01; n=2805), first flatus (MD=-15.08, 95%CI: -18.39, -11.76; *I*^2 =^ 96%; p < 0.01; n=3284), and bowel sounds recovery (MD=-10.96, 95%CI: -14.20, -7.72; *I*^2 =^ 94%; p < 0.01; n=2043). A subgroup analysis revealed that acupuncture not only reduced the duration of POI when administered alongside conventional care but also further expedited the recovery of gut function after colorectal surgery when integrated into the enhanced recovery after surgery (ERAS) pathway. The studies included in the analysis reported no instances of serious adverse events associated with acupuncture. We identified Zusanli (ST36), Shangjuxu (ST37), Neiguan (PC6), Sanyinjiao (SP6), Xiajuxu (ST39), Hegu (LI4), Tianshu (ST25), and Zhongwan (RN12) as primary acupoints for treating POI. Association rule mining suggested potential acupoint combinations including {ST37, ST39}≥{ST36}, {PC6, ST37}≥{ST36}, {SP6, ST37}≥{ST36}, and {ST25, ST37}≥{ST36}.

**Conclusion:**

Meta-analysis indicates acupuncture’s safety and superior effectiveness over postoperative care alone in facilitating gastrointestinal recovery. Machine-learning approaches highlight the importance of the lower He-sea points, including Zusanli (ST36) and Shangjuxu (ST37), in treating POI in CRC patients. Incorporating additional acupoints such as Neiguan (PC6) (for pain and vomiting) and Sanyinjiao (SP6) (for abdominal distension and poor appetite) can optimize treatment outcomes. These findings offer valuable insights for refining treatment protocols in both clinical and experimental settings, ultimately enhancing patient care.

## 1 Introduction

Postoperative ileus (POI) is an inevitable complication that detrimentally affects gastrointestinal function, resulting in delayed defecation and flatus, abdominal pain, nausea, vomiting, and intolerance to oral intake (1, 2). Despite the implementation of minimally invasive surgery and the enhanced recovery after surgery (ERAS), patients’ physical conditions have only shown limited improvement (3). In fact, some studies report durations of up to four days (4), leading to prolonged hospital stays and increased healthcare costs (5). Therefore, to enhance the postoperative recovery of the gastrointestinal system following colorectal surgery, additional measures are warranted.

Acupuncture, a non-pharmacological therapy, is gaining increasing attention as an effective treatment modality for perioperative medicine and gut motility disorders. Recent systematic reviews have reported promising outcomes of acupuncture in promoting the recovery of gastrointestinal function following surgery (6, 7). Ng et al. (8) observed that the time to first defecation after laparoscopic surgery for colorectal cancer (CRC) was 85.9 hours in the electroacupuncture (EA) group and 107.5 hours in the sham acupuncture (SA) group. In a recently published acupuncture trial, Wang et al. (9) demonstrated that EA reduced the time to first defecation (76.4 hours) compared to SA (90.0 hours) after laparoscopic CRC resection. These findings suggest that EA could be considered as a complementary therapy to the ERAS protocol to facilitate gastrointestinal function recovery.

Autonomic nervous system imbalance and inflammation play pivotal roles in the development of gastrointestinal dysmotility associated with POI (10–12). Accumulating evidence suggests that acupuncture can activate the vagal-adrenal pathway, leading to the regulation of gastrointestinal function and exerting anti-inflammatory effects (13). An experimental study (14) reported that acupuncture stimulation of the vagal nerve suppressed inflammation induced by intestinal manipulation in POI cases. This effect was achieved through the activation of the cholinergic anti-inflammatory pathway in macrophages, mediated by the α7 nicotinic acetylcholine receptor. A human study (15) also observed similar mechanisms, where transcutaneous electrical stimulation of acupoints on the lower limbs improved gastrointestinal function recovery by increasing plasma acetylcholine (Ach) and interleukin-10 (IL-10) levels while reducing levels of interleukin-6 (IL-6) and inducible nitric oxide synthase (iNOS). This improvement was associated with an increase in parasympathetic nerve activity and its anti-inflammatory effects.

The importance of acupoint combinations for successful acupuncture treatment is widely acknowledged. The selection of acupoints is guided by the principles of meridians, collaterals, and syndrome differentiation in traditional Chinese medicine (TCM) (16). However, there is currently no standardized protocol for acupoint selection in the treatment of POI in CRC.

Machine-learning approaches have garnered significant attention in acupuncture and Chinese medicine due to their broad applications. These strategies employ various methods such as association analysis, correlation analysis, classification, and clustering to analyze extensive datasets and identify meaningful patterns and relationships (17–20). In this paper, we first conducted a meta-analysis to assess the effectiveness of acupuncture for POI in CRC. Subsequently, we employed machine-learning techniques to analyze and explore patterns in the selection of acupuncture points in clinical practice for treating POI. The objective was to provide a guideline for establishing a standardized acupuncture treatment protocol for POI.

## 2 Materials and methods

### 2.1 Data sources and search strategy

A comprehensive search was conducted up to December 2022 without any language limitations, including eight databases (Cochrane Library, PubMed, Embase, Web of Science, SinoMed (CBM), China Journal Full-Text Database (CNKI), VIP, and WanFang Data). We used a combination of medical subject headings (MeSH) and keywords to develop retrieval strategies, including (“acupuncture” OR “electroacupuncture” OR “acupuncture therapy” OR “transcutaneous electrical acupoint stimulation” OR “acup*”) AND (“colorectal neoplasms” OR “colonic neoplasms” OR “rectal neoplasms”) AND (“postoperative ileus” OR “postoperative”). We adjusted the search terms for different databases accordingly.

### 2.2 Eligibility criteria

We selected randomized controlled trials (RCTs) to assess acupuncture’s efficacy in the treatment of POI in CRC. We applied the following inclusion criteria:

(1) Population: patients aged 18 years or older who were diagnosed with primary colorectal cancer and underwent colorectal resection.
(2) Intervention: manual acupuncture (MA), electroacupuncture (EA), or transcutaneous electrical acupoint stimulation (TEAS).
(3) Comparison: postoperative care (including conventional care or ERAS).
(4) Outcomes: recovery of gastrointestinal functions (such as time to first defecation and/or flatus and/or bowel sounds recovery), quality of life, or adverse events.

The following studies were excluded based on the exclusion criteria: (1) Patients who had a history of abdominal surgery, severe liver or kidney disease, severe heart failure, severe coagulation disorders, were taking drugs that affect bowel function within the past month, had a psychiatric disorder, had a history of drug or alcohol abuse, or were allergic to acupuncture needles. (2) Studies that employed interventions of auricular acupuncture, acupressure, moxibustion, acupoint embedding, acupoint injection, point application, or acupuncture combined with oral herbal medicine. (3) Repetitive publications, where data was extracted only from recent publications. (4) Review articles, meta-analysis, theoretical categorizations, or animal experiments. (5) Studies with unclear acupoint prescriptions that could not be extracted.

### 2.3 Data extraction

Relevant information was collected from eligible studies, including the first author’s name, publication date, sample size, type of cancer, type of surgery, interventions, acupoint prescriptions, and outcomes. Acupoint prescriptions were extracted using a “one group of main acupoints and one group of minor acupoints with one group of acupoint prescriptions” approach, following the standardized acupoint names according to the World Health Organization’s Standard Acupuncture Point Location in the Western Pacific Region (21). The data extraction process was conducted independently by two reviewers. In cases of missing or conflicting data, a third author was consulted to resolve discrepancies and achieve consensus.

### 2.4 Quality assessment

The revised Cochrane risk-of-bias tool (ROB 2) was utilized to assess the risk of bias in the included RCTs. The tool evaluated various aspects, including the randomization process, deviations from intended interventions, missing outcome data, measurement of the outcome, and selection of the reported results.

### 2.5 Machine-learning analysis

#### 2.5.1 Meta-analysis

A meta-analysis was conducted using R (version 4.2.1) to assess the effectiveness of acupuncture in treating POI in CRC patients. The mean difference (MD) along with a 95% confidence interval (CI) was utilized as the effect size for continuous data. Statistical significance was defined as a p-value less than 0.05. The presence of statistical heterogeneity was evaluated using Cochran’s Q test, and the magnitude of heterogeneity was assessed using Higgin’s and Thompson’s *I*^2^ statistic. If the *I*^2^ statistic was less than 50%, the results were pooled using a fixed effects model. However, if the *I*^2^ statistic exceeded 50%, a random-effects model was employed for the meta-analysis. Subgroup analysis was conducted to identify potential sources of substantial heterogeneity.

#### 2.5.2 Descriptive analysis

The extracted valid prescriptions were subjected to frequency analysis using Microsoft Excel 2019. Frequency statistics were collected on the application frequencies of acupoints, meridian acupoints, specific acupoints, and positioning distributions.

#### 2.5.3 Association rule mining analysis

For Apriori association rule analysis and chart generation, we utilized R (version 4.2.1) with the “arules” package and “arulesViz” package. The dataset was fitted using the “arules” package, and the charts were visualized using the “arulesViz” package. To enable comparison, we also utilized Python’s “PyFpgrowth” package to perform a frequent pattern growth algorithm.

The Apriori algorithm-based association rules consist of a left-hand-side (LHS) antecedent and a right-hand-side (RHS) consequence for each item in a list. These rules are derived based on three fundamental values: support, confidence, and lift. The support value measures the frequency of item sets occurring in the dataset, calculated by dividing the number of occurrences by the total number of records. Confidence represents the conditional probability of the RHS given the LHS, indicating the strength of the association between the two. Lift is a measure of the strength of the association rule, indicating how much more likely the RHS is to occur when the LHS is present, compared to when it is not. To identify important association rules, we considered multiple combinations of support and confidence thresholds. In this analysis, we examined the top 11 commonly used association rules based on 15% and 90% thresholds for support degree and confidence.

#### 2.5.4 Complex network analysis

To uncover connections among acupoints and identify acupoint combinations, we employed complex network analysis. Firstly, we converted the text information from the data source into a vector form and identified the acupoint names as the feature vector. We then input these vectors into a structured acupoints prescription table using a 0/1 format. Afterwards, we imported the aforementioned table into IBM SPSS Modeler (version 18.0) to generate an association rules flow that provided us with the associated acupoints and their respective weight values. Subsequently, we assigned a unique numerical identifier to each of the output nodes, and created a list of the node combinations and edges, which were then loaded into Cytoscape (version 3.9.1) to generate a network visualization of the associations.

#### 2.5.5 Correlation and cluster analysis

To explore the relationships between the top 10 most frequently selected acupoints, we performed a correlation analysis using R version 4.2.1. The “heatmap” package was employed to generate a correlation heatmap, providing visual insights into the associations among these acupoints. The correlation heatmap was constructed based on the Phi correlation coefficient, which measures the strength and direction of the relationship between two categorical variables. By analyzing the heatmap, we can identify patterns of correlation and assess the interconnections between the acupoints. Additionally, we employed cluster analysis to classify the relationships between the acupoints.

## 3 Result

### 3.1 Eligible studies and study characteristics

**Figure 1:**
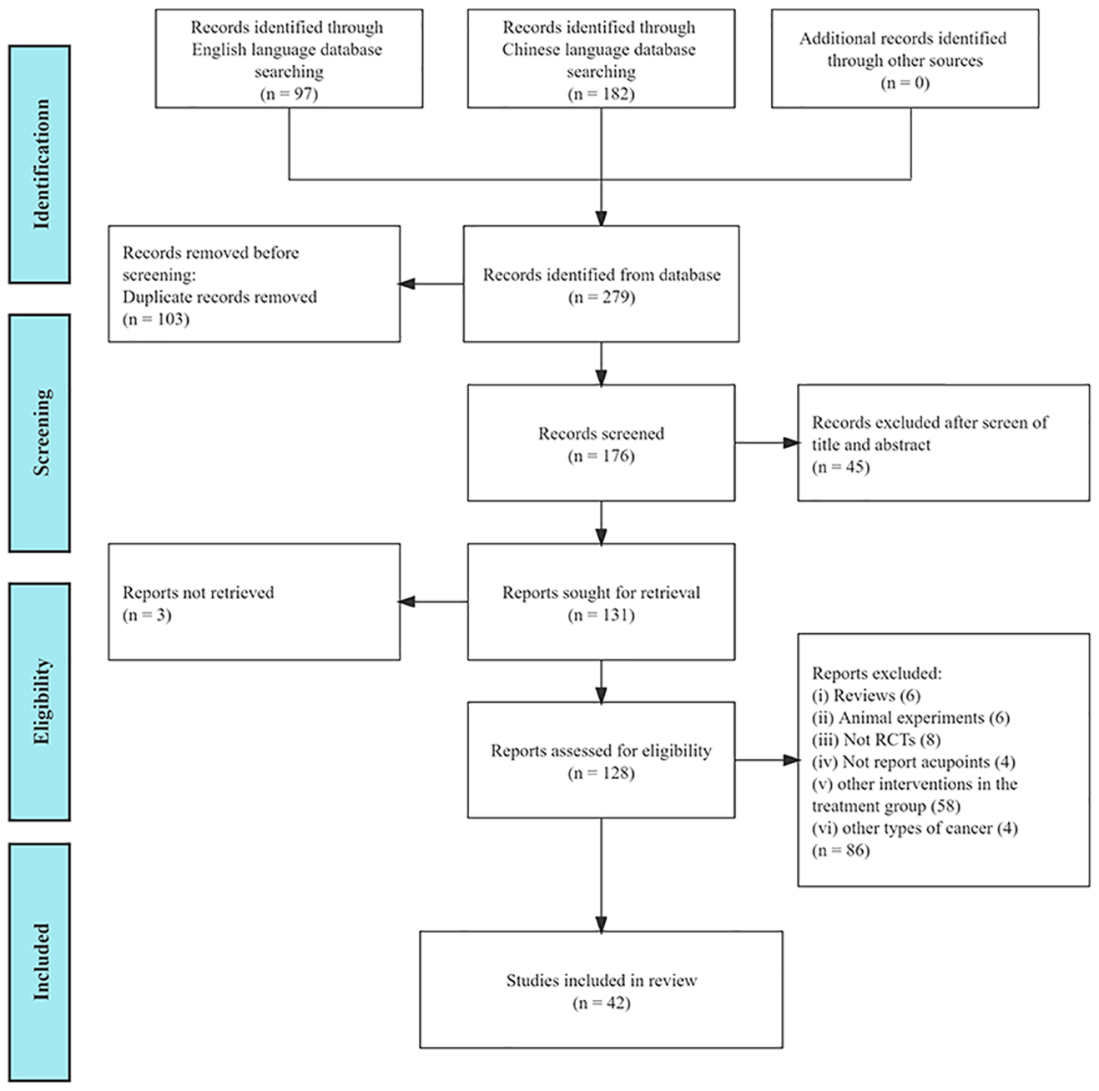
Flowchart of the study selection process.

A total of 279 relevant articles were identified through the search strategy described earlier. After removing duplicates, 176 papers underwent a screening process. Following the screening, 42 studies met the inclusion criteria and were included in our analysis (**Figure 1**). These included papers were published between 2006 and 2022, with 29 studies published within the last five years, representing 94% of the eligible studies. Detailed characteristics of the included studies can be found in **Table 1**.

**Table 1:** Characteristics of included studies.

Study (year)	Sample size (I/C), Gender (male/female), Age (I/C)	Type of cancer	Type of surgery	Intervetion	Control	Treatment duration	Outcomes
Wang Y et al. (2022) (9)	125/123, 153/95, 60.2 ± 11.0/60.2 ± 11.8	Colonrectal cancer	Laparoscopic	EA plus ERAS	ERAS	4 consecutive days after surgery	TFD, TFF, AE
Yang JW et al (2022) (22)	35/35, 48/22, 60.7 ± 12.7/60.7 ± 10.5	Colonrectal cancer	Laparoscopic	EA plus ERAS	ERAS	4 consecutive days after surgery or until discharge	TFD, TFF, AE
NG SSM et al (2013) (8)	55/55, 68/42, 67.4 ± 9.7/67.4 ± 10.7	Colonrectal cancer	Laparoscopic	EA plus CC	CC	4 consecutive days after surgery	TFD, TFF, AE
Zhang ZD et al (2014) (23)	19/20, 22/17, 63.0± 9.0/60.0 ± 10.0	Colonrectal cancer	Open	EA plus CC	CC	4 consecutive days after surgery	TFD, TFF, TBSR
Long Y et al (2021) (24)	30/30, 32/28, 63.5 ± 14.4/61.8 ± 11.9	Colonrectal cancer	NR	EA plus CC	CC	5 consecutive postoperative days or until passing flatus	TFD, TFF, TBSR
Zhu WJ et al (2021) (25)	43/43, 58/28, 61.0 ± 11.0/60.0 ± 12.0	Colon cancer	NR	EA plus ERAS	ERAS	5 consecutive postoperative days	TFD, TFF
Liang ZX et al (2020) (26)	60/60, 67/53, 59.5 ± 10.8/57.2 ± 12.2	Colonrectal cancer	NR	MA plus ERAS	ERAS	5 consecutive postoperative days	TFD, TFF, TBSR, QOL
Wang XZ et al (2022) (27)	30/30, 35/25, 66.1 ± 15.9/63.6 ± 11.6	Colonrectal cancer	Laparoscopic	MA plus CC	CC	5 consecutive postoperative days	TFD, TFF, TBSR, AE
Zhang K et al (2022) (28)	30/30, 30/30, NR	Colonrectal cancer	NR	MA plus CC	CC	3 consecutive postoperative days	TFD, TFF, TBSR
Li DX et al (2018) (29)	42/40, 49/33, 65.4 ± 13.3/65.0 ± 11.4	Colonrectal cancer	NR	MA plus ERAS	ERAS	Starting on postoperative day until the first defecation, first flatus, and first bowel sounds	TFD, TFF, TBSR
Xie JL et al (2014) (30)	45/45, 49/41, 59.5 ± 16.8/59.5 ± 16.8	Rectal cancer	NR	MA plus CC	CC	7 consecutive postoperative days	TFD, TFF
Tong WY et al (2014) (31)	42/42, 50/34, 58.6 ± 15.1/59.2± 14.7	Rectal cancer	Open	MA plus ERAS	ERAS	NR	TFD, TFF, TBSR
Zhang SY et al (2011) (32)	35/35, 52/18, NR	Colonrectal cancer	NR	MA plus CC	CC	10 consecutive postoperative days	TFD, TFF, TBSR
Wang Y et al (2019) (33)	20/20, 20/20, 62.5 ± 6.9/63.9 ± 6.9	Rectal cancer	NR	EA plus CC	CC	10 consecutive postoperative days	TFD, TFF, TBSR
Xue YL et al (2020) (34)	35/35, 36/34, 59.5 ± 13.8/63.5 ± 7.9	Colonrectal cancer	Laparoscopic	EA plus ERAS	ERAS	5 consecutive postoperative days	TFD, TFF, AE
Li Y et al (2017) (35)	30/30, 34/26, 65.8 ± 9.8/65.6 ± 8.4	Colonrectal cancer	Laparoscopic or open	EA plus CC	CC	5 consecutive postoperative days	TFD, TFF
Sun H et al (2021) (36)	42/42, 47/37, 59.1 ± 1.4/59.3 ± 1.6	Colonrectal cancer	NR	MA plus CC	CC	15 consecutive postoperative days	AE
Li WX et al (2021) (37)	40/40, 40/40, 61.0 ± 5.8/61.4 ± 6.5	Colonrectal cancer	Laparoscopic	EA plus CC	CC	From 30 min before anesthetic induction to the operation finished	TFD, TFF, TBSR
Yang JJ et al (2011) (38)	31/29, 39/23, 60.9 ± 6.6/62.0 ± 7.0	Colonrectal cancer	NR	EA plus CC	CC	Starting on postoperative day until the first defecation, then 3 consecutive days	TFD, TFF, TBSR
Zhou Y et al (2021) (39)	40/40, 46/34, 59.5 ± 10.5/59.3 ± 9.7	Colonrectal cancer	Laparoscopic	EA plus CC	CC	7 consecutive postoperative days	TFD, TFF, TBSR, AE
Xiao C et al (2014) (40)	30/30, 32/28, 55.9 ± 10.5/54.6 ± 10.3	Colonrectal cancer	Open	EA plus CC	CC	5 consecutive postoperative days	TFD, TFF, TBSR
Wang HM et al (2011) (41)	15/15, 20/10, 60.4 ± 11.0/58.0 ± 10.2	Colonrectal cancer	Open	MA plus ERAS	ERAS	5 consecutive postoperative days	TFD, TFF, TBSR
Xiao C et al (2016) (42)	12/12, 15/9, 60.7 ± 10.6/56.7 ± 13.7	Colonrectal cancer	NR	MA plus CC	CC	5 consecutive postoperative days	TFD, TFF
Si JG et al (2015) (43)	25/25, NR, NR	Colonrectal cancer	NR	EA plus CC	CC	Starting on postoperative day until the first flatus	TFF, TBSR
Niu CF et al (2008) (44)	16/16, 21/11, NR	Colonrectal cancer	NR	EA plus CC	CC	NR	TFF
Xiao L et al (2016) (45)	30/30, 33/27, 67.4 ± 16.4/68.5 ± 17.2	Colonrectal cancer	NR	MA plus CC	CC	14 consecutive postoperative days	TFF
Wang TY et al (2018) (46)	20/19, 21/18, 48.6 ± 6.1/51.4 ± 6.3	Colonrectal cancer	Open	EA plus CC	CC	2 consecutive preoperative days, 30 min before operation, 1 postoperative days	TFD, TFF
Mai SC et al (2017) (47)	20/20, 23/17, 52.0 ± 5.0/51.0 ± 1.0	Colonrectal cancer	Open	EA plus CC	CC	1 preoperative days, 30 min before operation	TFD, TFF, TBSR
Meng ZQ et al (2010) (48)	44/41, 47/38, 54.3/53.1	Colon cancer	NR	EA plus CC	CC	6 consecutive postoperative days or until the first bowel movement	TFD, TFF, QOL, AE
Gao W et al (2021) (15)	42/40, 49/33, 65.4 ± 13.3/65.0 ± 11.4	Colonrectal cancer	Laparoscopic or open	TEAS plus CC	CC	The first time was after operation, then 3 consecutive postoperative days	TFD, TFF, TBSR, AE
Feng DH et al (2020) (49)	28/32, 32/28, 66.3 ± 8.6/65.8 ± 5.7	Colon cancer	Laparoscopic	TEAS plus CC	CC	2 consecutive preoperative days, 30 min before operation	TFF
Wang D et al (2021) (50)	34/34, 47/21, 63.0 ± 7.6/62.8 ± 6.3	Colonrectal cancer	Laparoscopic	TEAS plus CC	CC	6 hours after operation, then 3 consecutive postoperative days	TFD, TFF
Huang W et al (2018) (51)	32/35, 37/30, 59.8 ± 11.6/59.1 ± 12.1	Colonrectal cancer	Laparoscopic	TEAS plus ERAS	ERAS	From 30 min before anesthetic induction to the operation finished	TFF
Cai WB et al (2021) (52)	47/47, 56/38, 56.5 ± 9.7/57.4 ± 10.4	Rectal cancer	Laparoscopic	TEAS plus CC	CC	From 30 min before anesthetic induction to the operation finished	TFF
Ren ZQ et al (2021) (53)	30/30, 35/25, 43.5 ± 6.2/43.1 ± 6.5	Rectal cancer	Laparoscopic	TEAS plus CC	CC	30 min before anesthetic induction and anesthetic resuscitation	TFD, TFF
Song YL et al (2020) (54)	46/46, 59/33, 61.5 ± 7.2/60.1 ± 6.8	Rectal cancer	Laparoscopic	TEAS plus CC	CC	From 30 min before anesthetic induction to the operation finished	TFD, TFF, TBSR
Xu YX et al (2006) (55)	30/30, 34/26, 63.3/58.6	Rectal cancer	NR	TEAS plus CC	CC	6 hours after operation, then Repeat treatment every 12 hours for 3consecutive days	TFD, TFF, TBSR
Yue S et al (2021) (56)	40/40, 45/35, 62.2 ± 7.5/63.8 ± 7.0	Colonrectal cancer	Laparoscopic	TEAS plus CC	CC	From 30 min before anesthetic induction to the operation finished	TFF
Fu HF et al (2022) (57)	25/21, 31/15, 62.0 ± 10.8/59.8 ± 10.6	Colonrectal cancer	Laparoscopic	TEAS plus CC	CC	From 30 min before anesthetic induction, lasting 30min	TFF, AE
Zhang HL et al (2020) (58)	45/45, 59/31, 64.0 ± 12.0/65.0 ± 11.0	Colonrectal cancer	NR	TEAS plus CC	CC	30 min after anesthetic resuscitation, then 3 consecutive postoperative days	TFF, TBSR
Xu B et al (2021) (59)	36/36, 37/35, 57.0 ± 4.1/56.9 ± 4.2	Colonrectal cancer	Laparoscopic	TEAS plus CC	CC	From 30 min before anesthetic induction to the operation finished	TFD, TFF, TBSR, AE
Wei QL et al (2019) (60)	52/52, 61/43, 60.2 ± 9.7/60.2 ± 9.8	Colonrectal cancer	Laparoscopic	TEAS plus CC	CC	Starting on postoperative day until the first defecation and first flatus	TFD, TFF, TBSR

I/C, Intervention group/Control group; MA, manual acupuncture; EA, electroacupuncture; TEAS, transcutaneous electrical acupoint stimulation; CC, conventional care; ERAS, enhanced recovery after surgery; TFD, time to first defecation; TFF, time to first flatus; TBSR, time to bowel sounds recovery; QOL, quality of life; AE, adverse events; NR, not reported.

### 3.2 Risk of bias assessment

**Figure 2:**
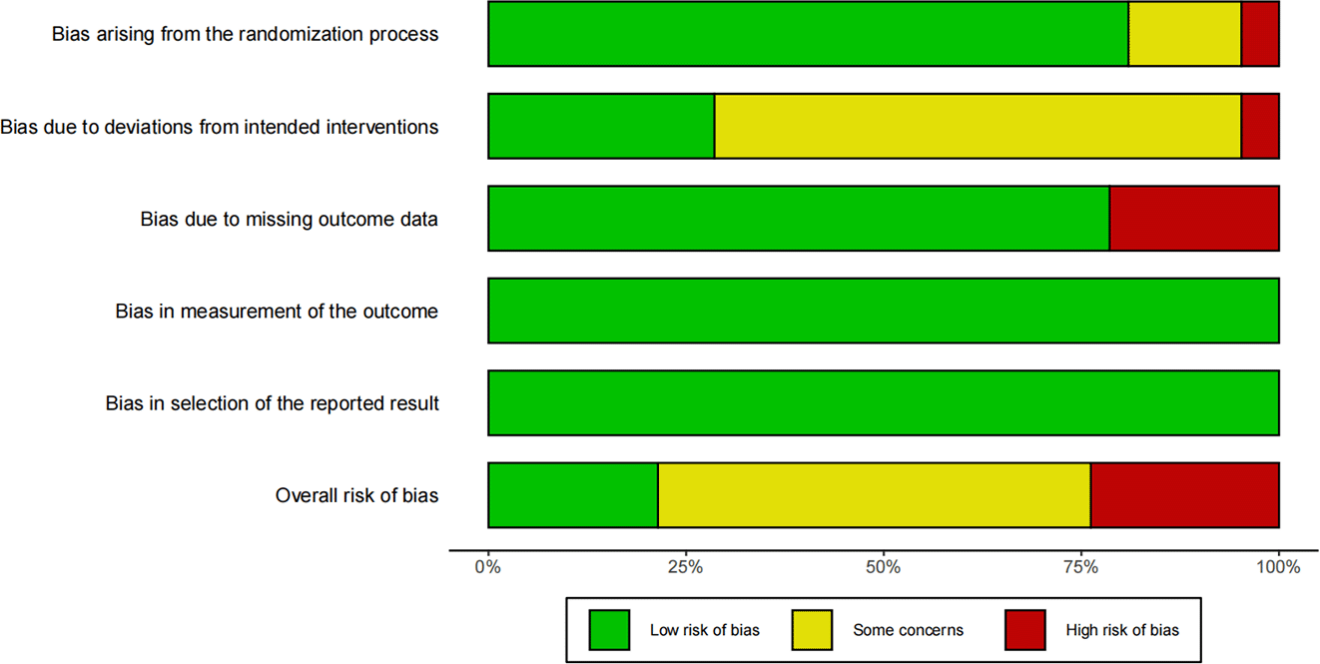
Risk of bias assessment.

In **Figure 2**, the bias assessments of the 42 included studies are presented. Among the retrieved studies, 36 studies provided clear descriptions of their randomized. However, in 9 studies, there was no description of the statistical analysis of missing outcome data, which may introduce potential bias. Furthermore, in many of the studies, there was uncertainty regarding the deviation from the intended intervention, potentially affecting the internal validity of the findings.

### 3.3 Meta-analysis

#### 3.3.1 The recovery of gastrointestinal function

##### 3.3.1.1 Overall effectiveness of acupuncture

**Figure 3:**
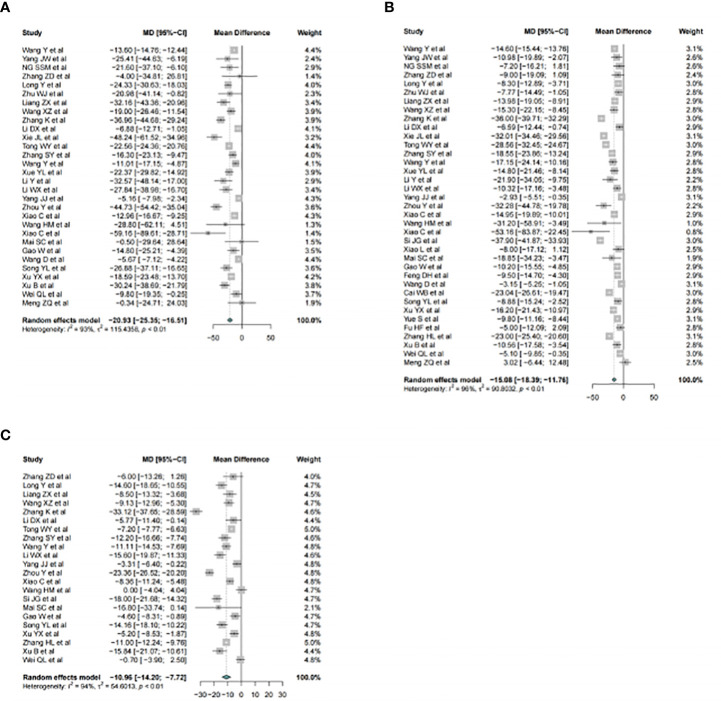
Forest plot of acupuncture therapy versus postoperative care alone for recovery of gastrointestinal function. Part **(A)** for time to first defecation. Part **(B)** for time to first flatus. Part **(C)** for time to bowel sounds recovery.

In the eligible studies, acupuncture therapy was found to be significantly effective in promoting the recovery of gastrointestinal function compared to postoperative care alone. The meta-analysis included 30 studies on acupuncture, which demonstrated a mean reduction of 20.93 hours in time to first defecation (TFD) in the test groups (MD=-20.93, 95%CI: -25.35, -16.51; *I*^2 =^ 93.0%; p < 0.01; n=2805) (**Figure 3A**). Furthermore, among the 37 RCTs that reported data on time to first flatus (TFF), there were significant reductions in the group treated with acupuncture (MD=-15.08, 95%CI: -18.39, -11.76; *I*^2 =^ 96%; p < 0.01; n=3284) (**Figure 3B**). Similarly, there were significant reductions in time to bowel sounds recovery (TBSR) in the intervention groups (MD=-10.96, 95%CI: -14.20, -7.72; *I*^2 =^ 94%; p < 0.01; n=2043) (**Figure 3C**), suggesting that acupuncture can facilitate bowel movements and promote the recovery of gastrointestinal function.

##### 3.3.1.2 Subgroup analysis

The subgroup analysis was conducted to further explore the effectiveness of acupuncture in promoting the recovery of gastrointestinal function based on different factors. The subgroups were defined based on: (I) type of acupuncture combined treatment: acupuncture plus conventional care or acupuncture plus ERAS; (II) type of cancer: colon cancer or rectal cancer; (III) type of surgery: laparoscopic surgery or open surgery; (IV) type of acupuncture modality: MA, EA, or TEAS. The results are listed in the **Table 2**.

**Table 2:** Subgroup analysis.

Outcomes	Subgroup	Studies	Effect Sizes MD	95%CI	Heterogeneity *I*^2^ (%)	p value
**Time to first defecation**	**Acupuncture combined treatment**					
	Acupuncture plus CC	22	-21.25	[-26.95, -15.55]	93	< 0.01
	Acupuncture plus ERAS	8	-19.62	[-26.03, -13.21]	92	< 0.01
	Test for subgroup differences: Q = 0.14, p = 0.71					
	**Cancer type**					
	Colon cancer	4	-19.86	[-25.68, -14.03]	34	< 0.01
	Rectal cancer	3	-26.52	[-47.27, -5.77]	93	< 0.01
	Test for subgroup differences: Q = 0.37, p = 0.54					
	**Surgery type**					
	Laparoscopic	11	-21.83	[-28.61, -15.06]	94	< 0.01
	Open	5	-16.56	[-24.51, -8.62]	83	< 0.01
	Test for subgroup differences: Q = 0.98, p = 0.32					
	**Acupuncture modality**					
	MA	9	-27.52	[-37.30, -17.75]	88	< 0.01
	EA	15	-18.98	[-25.15, -12.82]	87	< 0.01
	TEAS	6	-17.23	[-25.09, -9.37]	93	< 0.01
	Test for subgroup differences: Q = 2.86, p = 0.24					
**Time to first flatus**	**Acupuncture combined treatment**					
	Acupuncture plus CC	29	-15.16	[-19.14, -11.18]	97	< 0.01
	Acupuncture plus ERAS	8	-14.76	[-20.28, -9.23]	89	< 0.01
	Test for subgroup differences: Q = 0.01, p = 0.91					
	**Cancer type**					
	Colon cancer	3	-5.67	[-12.46, 1.13]	62	0.07
	Rectal cancer	6	-21.32	[-28.20, -14.45]	93	< 0.01
	Test for subgroup differences: Q = 10.09, p < 0.01					
	**Surgery type**					
	Laparoscopic	15	-11.51	[-14.81, -8.22]	92	< 0.01
	Open	5	-19.34	[-27.74, -10.95]	84	< 0.01
	Test for subgroup differences: Q = 2.90, p = 0.09					
	**Acupuncture modality**					
	MA	10	-22.06	[-29.62, -14.50]	94	< 0.01
	EA	16	-13.89	[-18.97, -8.81]	94	< 0.01
	TEAS	11	-11.47	[-15.67, -7.27]	95	< 0.01
	Test for subgroup differences: Q = 5.76, p = 0.06					
**Time to bowel sounds recovery**	**Acupuncture combined treatment**					
	Acupuncture plus CC	18	-12.24	[-15.91, -8.56]	94	< 0.01
	Acupuncture plus ERAS	4	-5.44	[-9.12, -1.76]	76	< 0.01
	Test for subgroup differences: Q = 6.55, p = 0.01					
	**Cancer type**					
	Colon cancer	0	–	–	–	–
	Rectal cancer	4	-9.21	[-12.95, -5.47]	83	< 0.01
	Test for subgroup differences: -					
	**Surgery type**					
	Laparoscopic	6	-13.10	[-19.32, -6.87]	95	< 0.01
	Open	5	-6.07	[-9.76, -2.38]	72	< 0.01
	Test for subgroup differences: Q = 3.62, p = 0.06					
	**Acupuncture modality**					
	MA	7	-10.82	[-18.58, -3.07]	96	< 0.01
	EA	10	-11.54	[-16.20, -6.87]	94	< 0.01
	TEAS	5	-10.00	[-14.30, -5.69]	85	< 0.01
	Test for subgroup differences: Q = 0.23, p = 0.89					

MA, manual acupuncture; EA, electroacupuncture; TEAS, transcutaneous electrical acupoint stimulation; CC, conventional care; ERAS, enhanced recovery after surgery; 95%CI, 95% confidence interval; MD, mean difference.

The subgroup analysis demonstrated that acupuncture, regardless of whether it was combined with postoperative conventional care or ERAS, was effective in promoting the recovery of gastrointestinal function. This suggests that acupuncture can be a beneficial adjunctive therapy in both treatment approaches. It is noteworthy that acupuncture combined with postoperative conventional care (MD=-12.24, 95%CI: -15.91, -8.56; *I*^2 =^ 94%; p < 0.01) is more effective than acupuncture combined with ERAS (MD=-5.44, 95%CI: -9.12, -1.76; *I*^2 =^ 76%; p < 0.01) in reducing TBSR, and this difference holds statistical significance (test for subgroup differences: Q = 6.55, p = 0.01). Regarding the type of cancer, it was found that acupuncture does not have statistically significant effects on reducing TFF in patients with colon cancer (MD=-5.67, 95%CI: -12.46, 1.13; *I*^2 =^ 62%; p = 0.07). However, acupuncture was found to be significantly effective in improving TFF in patients with rectal cancer (MD=-21.32, 95%CI: -28.20, -14.45; *I*^2 =^ 93%; p < 0.01). Due to the limited number of studies included, further validation should be conducted through adequately powered randomized trials to ensure sufficient statistical power and confirm these findings. The subgroup analysis based on the type of surgery indicated that acupuncture was beneficial for postoperative gastrointestinal function recovery in both laparoscopic surgery and open surgery patients. Additionally, the subgroup analysis showed that all types of acupuncture modalities had a significant effect in reducing TFF, TFD, and TBSR.

#### 3.3.2 Quality of life

Two trials reported on the outcomes of quality of life (QOL) in postoperative patients with CRC. Liang et al. (61) found that the combination of ERAS and acupuncture effectively improved postoperative quality of life using the European Organization for Research and Treatment of Cancer Quality of Life Questionnaire-Core 30 (EORTC QLQ-C30). On the other hand, Meng et al. (62) utilized the Quality of Life Status (QOLS) assessment tool based on the Edmonton Symptom Assessment System (ESAS) and reported no significant differences in quality of life measures between the acupuncture and control groups. Therefore, it is not possible to merge the effect sizes for these outcomes due to the lack of consistency between the assessment tools. And further clinical research is needed to validate whether acupuncture can improve the quality of life in postoperative patients.

#### 3.3.3 Safety

Out of the eleven studies that reported adverse events (AE) related to acupuncture, nine studies stated that no adverse events were observed. However, two studies (9, 22) reported mild adverse events that could potentially be associated with acupuncture use. These events included hematoma, pain, swelling, and residual needling sensation after needle removal. Importantly, these adverse events were self-limiting and did not require any specific medical interventions. It is worth noting that no serious adverse events were reported in any of the studies.

### 3.4 Acupoint distribution

**Figure 4:**
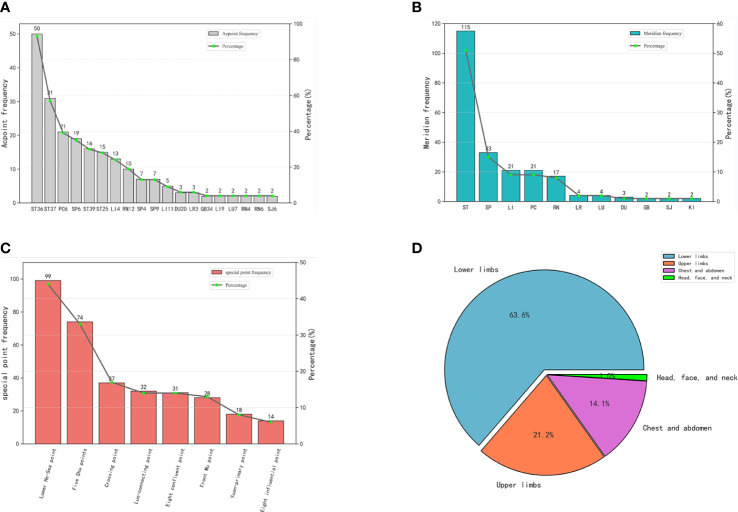
Acupoint distribution. Part **(A)** Frequency and percentage of acupoints. Part **(B)** Frequency and percentage of meridians. Part **(C)** Frequency and percentage of specific acupoints. Part **(D)** Frequency and percentage of site of acupoints.

According to the analysis of the 54 eligible acupoint prescriptions for the treatment of POI, a total of 31 acupoints were utilized. These acupoints were recorded a combined total of 224 occurrences. The top 10 frequently utilized acupoints comprised Zusanli (ST36, 93%), Shangjuxu (ST37, 57%), Neiguan (PC6, 39%), Sanyinjiao (SP6, 35%), Xiajuxu (ST39, 30%), Tianshu (ST25, 28%), Hegu (LI4, 24%), Zhongwan (RN12, 19%), Gongsun (SP4, 13%), and Yinlingquan (SP9, 13%) in descending order (**Figure 4A**). Zusanli (ST36) emerged as the most frequently employed acupoint, being present in 93% of the 54 acupoint prescriptions.

In the analyzed studies, a total of 31 distinct acupoints were utilized, and they were observed to be distributed across 11 meridians. These encompassed 8 regular meridians, along with the Conception Vessel (RN) and Governor Vessel (DU). However, it is noteworthy that the acupoint prescriptions did not incorporate the Bladder Meridian of Foot Taiyang (BL), the Heart Meridian of Hand Shaoyin (HT), and the Small Intestine Meridian of Hand Taiyang (SI). **Table 3** and **Figure 4B** illustrate the distribution of acupoints among various meridians. Among them, the Stomach Meridian of Foot Yangming (ST) exhibited the highest frequency, with a total occurrence of 115 instances. Subsequently, the Spleen Meridian of Foot Taiyin (SP) was utilized 33 times, followed by the Large Intestine Meridian of Hand Yangming (LI) and the Pericardium Meridian of Hand Jueyin (PC), both employed 21 times. In combination, these four meridians accounted for 84% of the overall utilization of acupoints.

**Table 3:** Frequency and percentage of meridians used for POI.

Meridians	Frequency	Percentage	Acupoint number	Acupoints (frequency)
ST	115	0.51	7	ST36 (50), ST37 (31), ST39 (16), ST25 (15), ST40 (1), ST41 (1), ST44 (1)
SP	33	0.15	3	SP6 (19), SP4 (7), SP9 (7)
LI	21	0.09	4	LI4 (13), LI11 (5), LI9 (2), LI10 (1)
PC	21	0.09	1	PC6 (21)
RN	17	0.08	6	RN12 (10), RN4 (2), RN6 (2), RN10 (1), RN13 (1), RN17 (1)
LR	4	0.02	2	LR3 (3), LR5 (1)
LU	4	0.02	3	LU7 (2), LU5 (1), LU9 (1)
DU	3	0.01	1	DU20 (3)
GB	2	0.01	1	GB34 (2)
SJ	2	0.01	1	SJ6 (2)
KI	2	0.01	2	KI3 (1), KI6 (1)

Among the total of 31 distinct acupoints, 28 of them were specific acupoints, with 12 possessing multiple attributes. Notably, Neiguan (PC6) was categorized as both the Luo-connecting acupoint and the eight confluent acupoint, while Zusanli (ST36) was identified as both the He-sea point and the lower He-Sea point. Remarkably, among the specific acupoints, the lower He-Sea points demonstrated a considerably higher frequency compared to other types, with a total occurrence of 99 instances. A detailed distribution of the specific acupoints is shown in **Table 4** and **Figure 4C**.

**Table 4:** Frequency and percentage of specific acupoints used for POI.

Specific acupoints	Frequency	Percentage	Acupoint number	Acupoints (frequency)
Lower He-Sea point	99	0.44	4	ST36 (50), ST37 (31), ST39 (16), GB34 (2)
Five Shu points	74	0.33	11	ST36 (50), SP9 (7), LI11 (5), LR3 (3), GB34 (2), SJ6 (2), KI3 (1), LU5 (1), LU9 (1), ST41 (1), ST44 (1)
Crossing point	37	0.17	7	SP6 (19), RN12 (10), DU20 (3), RN4 (2), KI6 (1), RN10 (1), RN13 (1)
Luo-connecting point	32	0.14	5	PC6 (21), SP4 (7), LU7 (2), LR5 (1), ST40 (1)
Eight confluent point	31	0.14	4	PC6 (21), SP4 (7), LU7 (2), KI6 (1)
Front Mu point	28	0.13	4	ST25 (15), RN12 (10), RN4 (2), RN17 (1)
Yuan-primary point	18	0.08	4	LI4 (13), LR3 (3), KI3 (1), LU9 (1)
Eight influential point	14	0.06	4	RN12 (10), GB34 (2), LU9 (1), RN17 (1)

Moreover, when considering the selection of acupoint application, the lower limbs emerged as the most frequently chosen area, constituting approximately 63% of the overall frequency (**Table 5**, **Figure 4D**).

**Table 5:** Frequency and percentage of site of acupoints used for POI.

Site of acupoints	Frequency	Percentage	Acupoint number	Acupoints (frequency)
Lower limbs	141	0.63	14	ST36 (50), ST37 (31), ST 39 (16), ST40 (1), ST41 (1), ST44 (1), SP6 (19), SP4 (7), SP9 (7), LR3 (3), LR5 (1), GB34 (2), KI3 (1), KI6 (1)
Upper limbs	48	0.21	9	LI4 (13), LI11 (5), LI9 (2), LI10 (1), PC6 (21), LU7 (2), LU5 (1), LU9 (1), SJ6 (2)
Chest and abdomen	32	0.14	7	ST25 (15), RN12 (10), RN4 (2), RN6 (2), RN10 (1), RN13 (1), RN17 (1)
Head, face, and neck	3	0.01	1	DU20 (3)

### 3.5 Association rule mining analysis

#### 3.5.1 Apriori algorithm-based association rule analysis

**Figure 5:**
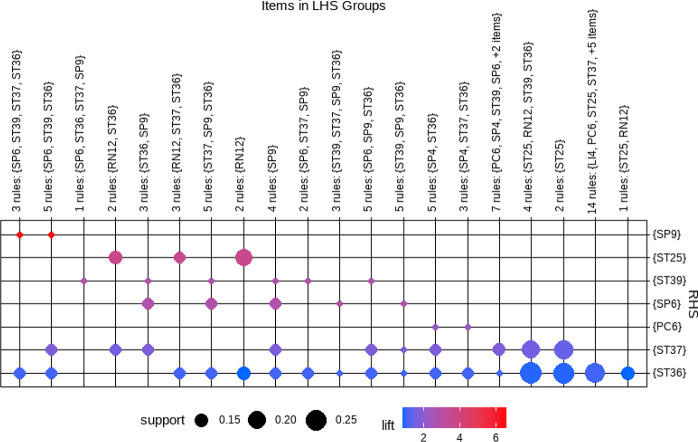
Grouped matrix for 79 Apriori algorithm-based association rules.

Through the analysis of the acupuncture prescription, a total of 79 association rules were generated. The overall distribution of these association rules is depicted in **Figure 5**, utilizing a grouped matrix diagram. In this representation, circles that appear redder in color indicate a higher degree of lift, while circles with larger sizes indicate greater support.

The implementation of the Apriori algorithm was carried out using R software, employing a minimum support threshold of 15% and a minimum confidence threshold of 90%. As a result, a total of 11 commonly observed acupoint combinations were identified, including: {ST37}≥{ST36}, {PC6}≥{ST36}, {SP6}≥{ST36}, {ST36, ST39}≥{ST37}, {ST37, ST39}≥{ST36}, {LI4}≥{ST36}, {SP6, ST37}≥{ST36}, {ST25, ST37}≥{ST36}, {PC6, ST37}≥{ST36}, {ST25, ST36}≥{ST37}, and {RN12}≥{ST25}. **Table 6** furnishes comprehensive information regarding the support, confidence, and lift of the identified acupoint association rules. Notably, upon analysis, it was determined that all 11 association rules displayed lift values greater than 1. This finding signifies that the selected acupoints, in conjunction with their respective antecedents, exhibited a higher likelihood of being utilized together compared to selecting the consequent acupoint in isolation.

**Table 6:** The top 11 Apriori algorithm-based association rules.

No.	Association rules	Support	Confidence	Lift
1	{ST37} ≥ {ST36}	0.57	1.00	1.08
2	{PC6} ≥ {ST36}	0.39	1.00	1.08
3	{SP6} ≥ {ST36}	0.35	1.00	1.08
4	{ST36, ST39} ≥ {ST37}	0.26	1.00	1.74
5	{ST37, ST39} ≥ {ST36}	0.26	1.00	1.08
6	{LI4} ≥ {ST36}	0.24	1.00	1.08
7	{SP6, ST37} ≥ {ST36}	0.24	1.00	1.08
8	{ST25, ST36} ≥ {ST37}	0.22	0.92	1.61
9	{ST25, ST37} ≥ {ST36}	0.22	1.00	1.08
10	{PC6, ST37} ≥ {ST36}	0.22	1.00	1.08
11	{RN12} ≥ {ST25}	0.19	1.00	3.60

#### 3.5.2 Frequent pattern growth algorithm-based association rule analysis

**Tables 7**, **8** present a summary of the outcomes obtained from the frequent pattern growth algorithm and the Frequent pattern growth algorithm-based association rules, respectively, for the eligible RCTs. These results demonstrate a remarkable consistency with those obtained through the application of the Apriori algorithm-based association rules. Such concurrence underscores the reliability and reproducibility of our findings.

**Table 7:** Frequent patterns of acupoints used on acupuncture treatment for POI.

No.	Frequent pattern	Support
1	ST36, ST37	0.57
2	ST36, PC6	0.39
3	ST36, SP6	0.35
4	ST37, ST39	0.26
5	ST36, ST39	0.26
6	ST36, ST37, ST39	0.26
7	ST36, ST25	0.24
8	ST37, SP6	0.24
9	ST36, ST37, SP6	0.24
10	LI4, ST36	0.24
11	ST37, ST25	0.22
12	ST36, ST37, ST25	0.22
13	ST37, PC6	0.22
14	ST36, ST37, PC6	0.22
15	RN12, ST25	0.19

**Table 8:** Frequent pattern growth algorithm-based association rules for POI treatment.

No.	Antecedents	Consequents	Antecedent support	Consequent support	Support
1	{ST37}	{ST36}	0.57	0.93	0.57
2	{PC6}	{ST36}	0.39	0.93	0.39
3	{SP6}	{ST36}	0.35	0.93	0.35
4	{ST37, ST39}	{ST36}	0.26	0.93	0.26
5	{ST39, ST36}	{ST37}	0.26	0.57	0.26
6	{LI4}	{ST36}	0.24	0.93	0.24
7	{SP6, ST37}	{ST36}	0.24	0.93	0.24
8	{ST37, PC6}	{ST36}	0.22	0.93	0.22
9	{ST25, ST37}	{ST36}	0.22	0.93	0.22
10	{ST36, ST25}	{ST37}	0.24	0.57	0.22
11	{RN12}	{ST25}	0.19	0.28	0.19

### 3.6 Complex network analysis

**Figure 6:**
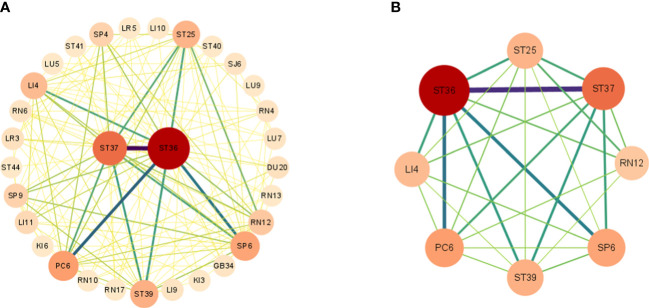
Complex network analysis. Part **(A)** Complex network analysis of acupuncture for POI treatment. Part **(B)** Acupoints with a degree greater than 14.

Through complex network analysis (**Figure 6**), a total of 31 acupoints and 144 edge weights were derived. Notably, eight acupoints demonstrated a degree exceeding 14, including Zusanli (ST36), Shangjuxu (ST37), Neiguan (PC6), Sanyinjiao (SP6), Xiajuxu (ST39), Hegu (LI4), Tianshu (ST25), and Zhongwan (RN 12). Among these acupoints, Zusanli (ST36) and Shangjuxu (ST37) were the most frequently employed, thereby signifying their significance as the core components of acupuncture point combinations. Importantly, the outcomes obtained from the complex network analysis align consistently with the results derived from the association rule analysis.

### 3.7 Correlation and cluster analysis

**Figure 7:**
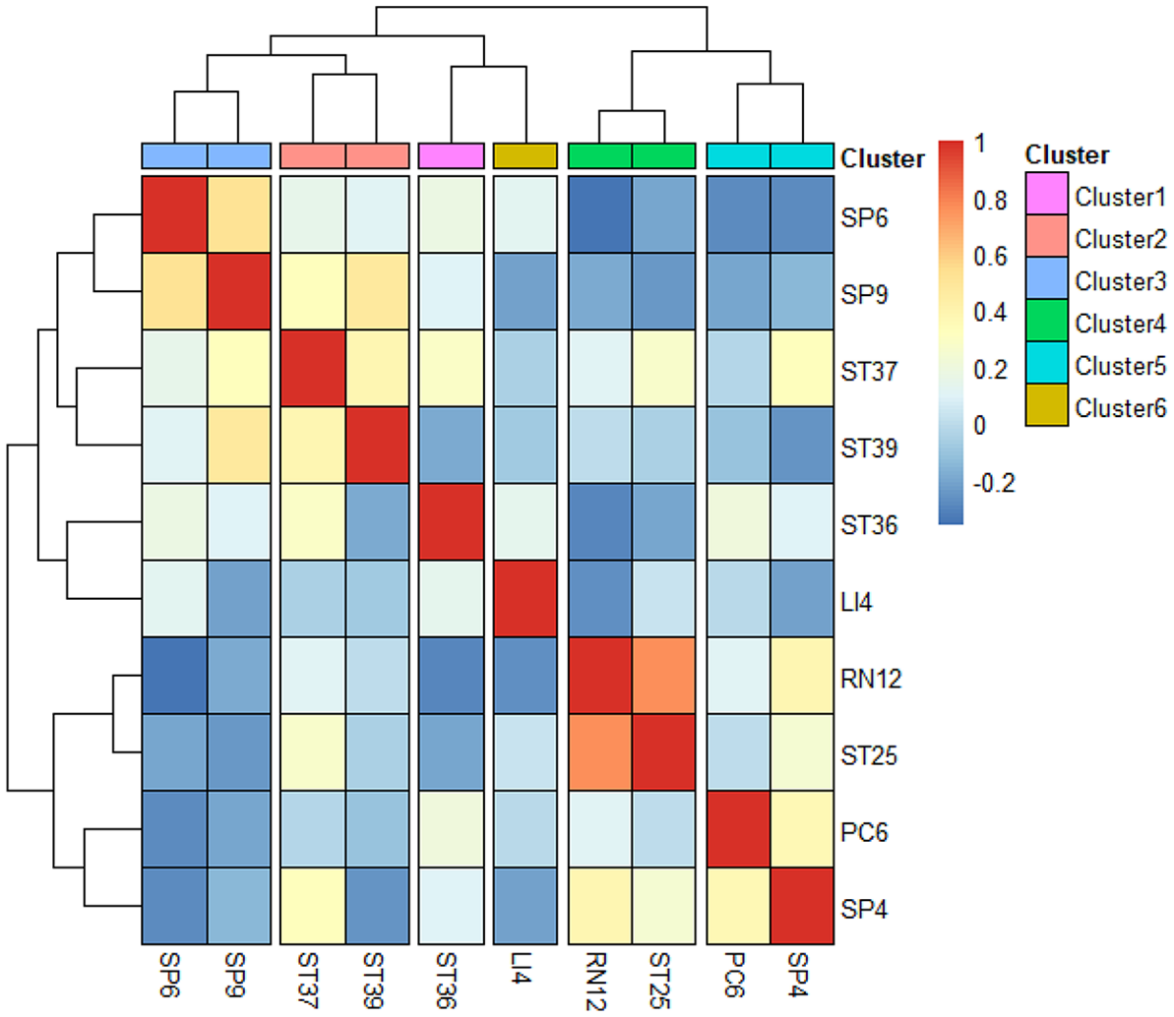
Correlation and cluster analysis of the top 10 frequently selected acupoints.

A correlation heatmap generated to help us derive effective treatment directions and principles for acupuncture treatment of POI. The top 10 frequently selected acupoints were then grouped into six major clusters based on their degree of correlation with each other: Cluster 1 - Zusanli (ST36), Cluster 2 - Shangjuxu (ST37) and Xiajuxu (ST39), Cluster 3 - Sanyijiao (SP6) and Yinlingquan (SP9), Cluster 4 - Zhongwan (RN12) and Tianshu (ST25), Cluster 5 - Neiguan (PC6) and Gongsun (SP4), and Cluster 6 - Hegu (LI4) (**Figure 7**).

## 4 Discussion

In this study, a meta-analysis was conducted to investigate the efficacy of acupuncture intervention in promoting the recovery of gastrointestinal function in patients after undergoing colorectal cancer surgery. The results indicated that acupuncture intervention was associated with superior effectiveness in several key outcomes, including TFD, TFF, and TBSR.

In the subgroup analysis, we made some noteworthy observations. It was found that acupuncture, when combined with postoperative conventional care, is more effective in reducing TBSR compared to its combination with the ERAS pathway. The ERAS pathway, which incorporates the preventive use of antibiotics, perioperative multimodal analgesia, avoidance of opioid use, early removal of urinary catheters and nasogastric tubes, early mobilization, and nutritional support, has been increasingly utilized. Patients undergoing this pathway experienced less pain, lower opioid requirements, and a shorter recovery time. We speculate that the observed phenomenon may be attributed to the fact that the combined ERAS protocal could have already mitigated the surgical stress response to a certain extent, leaving limited room for further improvement in clinical outcomes by acupuncture intervention.

Furthermore, in the subgroup analysis focusing on different types of cancer, it was discovered that acupuncture does not yield statistically significant effects in reducing TFF for patients with colon cancer. However, it was found that acupuncture demonstrates a significant effectiveness in improving TFF for patients with rectal cancer. This discrepancy may be attributed to the greater importance of the colon in gastrointestinal motility compared to the rectum. Therefore, we hypothesize that patients who have undergone rectal resection are more likely to derive benefits from acupuncture therapy.

The studies included in the analysis demonstrated that there were no serious AE related to acupuncture. However, further verification is still necessary to determine whether acupuncture can effectively improve the overall quality of life in postoperative patients.

Additionally, we employed data mining to identify the most effective acupoint combinations for treating POI in CRC. Zusanli (ST36) was identified as the most used acupoint of all prescriptions evaluated in this study. This acupoint, located on the anterior aspect of the leg, is classified as both the He-sea point of the Stomach Meridian of Foot Yangming and the lower He-sea point of the stomach. Numerous studies have reported the diverse efficacies of acupuncture at Zusanli (ST36). These include its anti-inflammatory effects, ability to enhance the immune system, and promotion of postoperative gastrointestinal function restoration (63–65). In a POI model, acupuncture at Zusanli (ST36) was found to decrease the concentration of inflammatory cytokines tumor necrosis factor-α (TNF-α) and IL-6 in the serum. It also suppressed local intestinal inflammation by inhibiting the infiltration of macrophages and neutrophils in the intestine (66). Acupuncture at Zusanli (ST36) has also shown the potential to repair damage to interstitial cells of Cajal (ICCs), which play a critical role in regulating gastrointestinal motility (67). Recent studies have demonstrated the unique ability of EA at Zusanli (ST36) to modulate endotoxin-induced systemic inflammation through its impact on the sympathetic pathways (68). Shangjuxu (ST37), classified as the lower He-sea point of the large intestine, is commonly used in acupuncture. Clinical studies show that acupuncture at Shangjuxu (ST37) effectively alleviates visceral hypersensitivity and reduces pain perception in internal organs (69).

Acupuncturists often combine multiple acupoints to effectively address the symptoms of a specific disease, aiming to achieve enhanced therapeutic outcomes. In our study, through association rule mining, we identified that the primary acupoints for POI treatment are Zusanli (ST36), Shangjuxu (ST37), Neiguan (PC6), Sanyinjiao (SP6), Xiajuxu (ST39), Hegu (LI4), Tianshu (ST25), and Zhongwan (RN12). Among these, the acupuncture points Zusanli (ST36) and Shangjuxu (ST37) can be regarded as the fundamental acupoints in the combinations used to treat POI.

By using a clustering algorithm, the top 10 frequently selected acupoints were clustered into six major clusters, signifying the primary treatment principles, including the regulation of gastrointestinal function, the alleviation of pain and vomiting, and the strengthening of the spleen. In TCM, POI is characterized as a disorder of intestinal conduction. The pathogenesis of POI is mainly due to the upward reversal of stomach-qi and obstruction of fu-qi, which matches the treatment direction we have concluded. The first 2 clusters, including Zusanli (ST36), Shangjuxu (ST37), and Xiajuxu (ST39), which can promote bowel function and remove stagnation, were the main acupoints in treating POI. The other 4 clusters were defined as preliminary adjunct acupoints. Cluster 3, consisting of Sanyinjiao (SP6) and Yinlingquan (SP9), can strengthen the spleen and invigorate blood to improve abdominal distension and poor appetite. Cluster 4, consisting of Tianshu (ST25) and Zhongwan (RN12), located on the abdomen as the “Front Mu point” of the large intestine and stomach respectively, can be applied to treat deficiency of the stomach and intestine. Cluster 5, consisting of Neiguan (PC6) and Gongsun (SP4), known as the pain-relieving and anti-emetic acupoints, as the “Luo-connecting point” of the pericardium and spleen, can be used to alleviate pain and vomiting. Finally, Cluster 6, Hegu (LI4) can be used to harmonize gastrointestinal function.

The lower He-sea points, which include Zusanli (ST36), Shangjuxu (ST37), and Xiajuxu (ST39), are acupoints commonly employed in the treatment of POI. These specific acupoints account for 44% of the total frequency of acupoint usage in POI treatment. According to the ancient Chinese literature “Curing Viscera Diseases by the lower He-sea points” in Lingshu Jing, these points, located on the lower limbs, are where the qi of the six-fu organs converges. They have been historically associated with the treatment of gastrointestinal dysfunctions and are often selected as primary acupoints for addressing digestive symptoms. Moreover, the selection of the lower He-sea points on the lower limb for acupuncture treatment of POI offers advantages in terms of tolerability and safety, particularly for patients who have undergone abdominal surgery. Many patients may experience discomfort in the abdominal region, and needling acupuncture points in this area may increase their fear and anxiety. By targeting the lower He-sea points, studies have demonstrated beneficial effects in reducing the time to first flatus and defecation, as well as decreasing the incidence of postoperative abdominal pain (70–72). The therapeutic effects of stimulating the lower He-sea points are attributed to their regulatory impact on the immune system, reduction of inflammation, and alleviation of gastrointestinal mucosal lesions in models of acute mucosal lesions. This mechanism of action involves the modulation of serum concentrations of interleukin-1β (IL-1β) and high mobility group protein B1 (HMGB1), as well as the suppression of nicotinic acetylcholine receptor α7 (nAChR α7) and nuclear factor-kappa B (NF-κB) expression in mucosal tissue (73–75).

In recent years, there has been a continuous exploration of the profound mechanisms underlying the therapeutic effects of acupuncture for the treatment of POI. It is widely recognized that acupuncture exerts regionally specific effects (76, 77), thus raising the important question of whether the site of acupoint stimulation, such as those located in the limb area or abdominal region, can influence the outcomes of POI. Generally, it is postulated that proximal acupoint stimulation may yield more favorable results. However, the majority of existing research primarily focuses on limb acupoints, particularly those situated in the lower extremities, which aligns with the selection of acupoint distribution in our study. Acupuncture therapy operates on the fundamental principle that the stimulation of specific points on the body, known as acupoints, can engender therapeutic effects in distant areas of the body. A randomized controlled study conducted across multiple centers has provided evidence that TEAS administered to the lower extremities can effectively reduce the incidence of POI and expedite the recovery of gastrointestinal function (15). Furthermore, recent investigations have demonstrated that EA applied the acupoint Zusanli (ST36), rather than Tianshu (ST25), exhibits a significant capacity to enhance bowel function recovery in patients who have undergone laparoscopic elective colorectal resection for CRC (22). Additionally, the specificity of acupuncture’s effects on distinct body regions has garnered support from various animal studies. Notably, in a model of systemic inflammation, it was observed that electrical acupuncture applied to Zusanli (ST36), but not Tianshu (ST25), can activate the vagal-adrenal anti-inflammatory axis in septic mice (68). Recent investigations have shed light on the neuroanatomical basis underlying the specificity of acupuncture points in modulating the vagal-adrenal axis (13). Specifically, these studies have demonstrated that PROKR2-marked dorsal root ganglia sensory neurons innervate the deep fascia of the hindlimb but not the abdominal fascia, playing a crucial role in mediating the effects of low-intensity, deep stimulation at the Zusanli (ST36) acupoint on the vagal-adrenal axis. However, it has been revealed that high-intensity EA stimulation at both Tianshu (ST25) and Zusanli (ST36) acupoints can activate spinal sympathetic reflexes independently of PROKR2ADV neurons, leading to anti-inflammatory responses that are not reliant on vagal efferents. Thus, it appears that acupoints situated in the abdomen or limbs may exert their effects through distinct autonomic pathways. Furthermore, an additional study has provided evidence that the concurrent stimulation of abdominal acupoints, specifically Tianshu (ST25) and Zhongwan (RN12), along with limb acupoints like Shanglian (LI9) and Xiajuxu (ST39), demonstrates superior effectiveness in enhancing the recovery of intestinal function following surgery for rectal cancer, surpassing the outcomes achieved by stimulating these acupoints individually (33).

The regionally specific effects of acupuncture points are closely linked to the distribution of neural anatomy and can be influenced by the intensity of stimulation and the depth at which the needle is inserted (78, 79). However, it is noteworthy that while most mechanism studies concentrate on individual acupuncture points, clinical practice often involves the use of combinations of acupoints. Hence, it is imperative to delve deeper into the underlying mechanisms that may explain potential synergistic or interactive effects among different acupuncture points. Particularly, it would be valuable to ascertain whether different acupoints collaborate in a complementary or antagonistic manner, shedding light on their intricate interplay.

Several limitations should be acknowledged in relation to this study. Firstly, it is important to recognize that the overall quality of the included publications exhibited a bias towards moderate to high risk. Achieving complete double-blindness in acupuncture treatment studies is inherently challenging, and a number of studies did not provide adequate descriptions of statistical analysis for missing outcome data, leading to a potential risk of bias. Future investigations on acupuncture for POI should strive for greater rigor to enhance the quality of the evidence. Secondly, factors such as stimulation intensities and treatment time (including duration and frequency) play a crucial role in determining the clinical efficacy of acupoint specificity. As indicated by our meta-analysis, considerable heterogeneity was observed among the included studies. However, these factors were not subjected to detailed analysis within this study. Thirdly, the identification of potential acupoint combinations using machine-learning techniques is a result of data integration. Given that different acupoint treatments may engage distinct physiological pathways, further animal experiments and clinical trials are warranted to investigate whether the combination of different acupoints elicits synergistic or antagonistic effects. Lastly, it is crucial to acknowledge and address the potential limitations and risks associated with acupuncture. The effectiveness of acupuncture is heavily dependent on the skill and experience of the acupuncturist. Improperly performed acupuncture by untrained or inexperienced practitioners can lead to tissue damage and may provide limited clinical benefits. Moreover, the use of needles in acupuncture can pose challenges for individuals with needle phobia, potentially impeding their willingness to undergo treatment. When administering acupuncture, healthcare providers should take into consideration patients’ individual preferences, acceptance levels, and the availability of alternative treatment modalities. This will enable them to make informed decisions and provide thoughtful recommendations regarding the appropriateness of acupuncture as a therapeutic option.

## 5 Conclusion

The results of the meta-analysis demonstrated that acupuncture therapy was significantly more effective than postoperative care alone in facilitating the recovery of gastrointestinal function in patients after undergoing colorectal cancer surgery. Furthermore, no serious adverse events related to acupuncture were identified in the analyzed studies. Our investigation revealed that acupoints located in the lower limbs, particularly the lower He-sea points, played a crucial role in the treatment of POI. The underlying mechanism by which acupuncture improves POI involves the regulation of autonomic nervous system imbalances and the induction of anti-inflammatory effects. Considering its safety, convenience, proven effectiveness, and generally well-tolerated nature, acupuncture emerges as a promising non-pharmacological approach for perioperative management.

## Data availability statement

The original contributions presented in the study are included in the article/supplementary material. Further inquiries can be directed to the corresponding author.

## Author contributions

XZ designed the strategy of this research and wrote the manuscript. WY, LS and JS participated into analysis implementation and organized discussion of the results. WY, XZ and WD implemented the strategy with software, and analyzed the data. JS dedicated in results analysis and manuscript revision. GY and LT have polished this paper with professional perspective and put forward some constructive suggestion to the data analysis process. All authors read and approved the final manuscript.

## References

[1] Sommer NP Schneider R Wehner S Kalff JC Vilz TO. State-of-the-art colorectal disease: postoperative ileus. Int J Colorectal Dis (2021) 36(9):2017–25. doi: 10.1007/s00384-021-03939-1 PMC8346406 33977334

[2] Vather R Trivedi S Bissett I. Defining postoperative ileus: results of a systematic review and global survey. J Gastrointest Surg (2013) 17(5):962–72. doi: 10.1007/s11605-013-2148-y 23377782

[3] Lee SM Kang SB Jang JH Park JS Hong S Lee TG. Early rehabilitation versus conventional care after laparoscopic rectal surgery: a prospective, randomized, controlled trial. Surg Endosc. (2013) 27(10):3902–9. doi: 10.1007/s00464-013-3006-4 23708720

[4] Veldkamp R Kuhry E Hop WC Jeekel J Kazemier G Bonjer HJ. Laparoscopic surgery versus open surgery for colon cancer: short-term outcomes of a randomised trial. Lancet Oncol (2005) 6(7):477–84. doi: 10.1016/S1470-2045(05)70221-7 15992696

[5] van Bree SH Nemethova A Cailotto C Gomez-Pinilla PJ Matteoli G Boeckxstaens GE. New therapeutic strategies for postoperative ileus. Nat Rev Gastroenterol Hepatol (2012) 9(11):675–83. doi: 10.1038/nrgastro.2012.134 22801725

[6] Cheong KB Zhang JP Huang Y. The effectiveness of acupuncture in postoperative gastroparesis syndrome-a systematic review and meta-analysis. Complement Ther Med (2014) 22(4):767–86. doi: 10.1016/j.ctim.2014.05.002 25146082

[7] Liu Y May BH Zhang AL Guo X Lu C Xue CC. Acupuncture and related therapies for treatment of postoperative ileus in colorectal cancer: A systematic review and meta-analysis of randomized controlled trials. Evid Based Complement Alternat Med (2018) 2018:3178472. doi: 10.1155/2018/3178472 30151019 PMC6087601

[8] Ng SSM Leung WW Mak TWC Hon SSF Li JCM Wong CYN. Electroacupuncture reduces duration of postoperative ileus after laparoscopic surgery for colorectal cancer. Gastroenterology (2013) 144(2):307–313.e1. doi: 10.1053/j.gastro.2012.10.050 23142625

[9] Wang Y Yang JW Yan SY Lu Y Han JG Pei W. Electroacupuncture vs sham electroacupuncture in the treatment of postoperative ileus after laparoscopic surgery for colorectal cancer: A multicenter, randomized clinical trial. JAMA Surg (2023) 158(1):20–7. doi: 10.1001/jamasurg.2022.5674 PMC9631228 36322060

[10] Boeckxstaens GE de Jonge WJ. Neuroimmune mechanisms in postoperative ileus. Gut (2009) 58(9):1300–11. doi: 10.1136/gut.2008.169250 19671558

[11] de Jonge WJ van der Zanden EP The FO Bijlsma MF van Westerloo DJ Bennink RJ. Stimulation of the vagus nerve attenuates macrophage activation by activating the Jak2-STAT3 signaling pathway. Nat Immunol (2005) 6(8):844–51. doi: 10.1038/ni1229 16025117

[12] Farro G Gomez-Pinilla PJ Di Giovangiulio M Stakenborg N Auteri M Thijs T. Smooth muscle and neural dysfunction contribute to different phases of murine postoperative ileus. Neurogastroenterol Motil. (2016) 28(6):934–47. doi: 10.1111/nmo.12796 26891411

[13] Liu S Wang Z Su Y Qi L Yang W Fu M. A neuroanatomical basis for electroacupuncture to drive the vagal-adrenal axis. Nature (2021) 598(7882):641–5. doi: 10.1038/s41586-021-04001-4 PMC9178665 34646018

[14] Yang NN Yang JW Ye Y Huang J Wang L Wang Y. Electroacupuncture ameliorates intestinal inflammation by activating α7nAChR-mediated JAK2/STAT3 signaling pathway in postoperative ileus. Theranostics (2021) 11(9):4078–89. doi: 10.7150/thno.52574 PMC7977469 33754049

[15] Gao W Li W Yan Y Yang R Zhang Y Jin M. Transcutaneous electrical acupoint stimulation applied in lower limbs decreases the incidence of paralytic ileus after colorectal surgery: A multicenter randomized controlled trial. Surgery (2021) 170(6):1618–26. doi: 10.1016/j.surg.2021.08.007 34497027

[16] Chen Q Liang FX Wu S Lu W Wang H. Theoretic exploration and clinical application of acupoint combination based on biaoben theory. Zhongguo Zhen Jiu. (2018) 38(5):5053–9. doi: 10.13703/j.0255-2930.2018.05.015 29797915

[17] Dai N. Analysis of Data Interaction Process Based on data mining and Neural Network Topology Visualization. Comput Intell Neurosci (2022) 2022:1817628. doi: 10.1155/2022/1817628 35814595 PMC9259330

[18] Wang Y Shi X Efferth T Shang D. Artificial intelligence-directed acupuncture: a review. Chin Med (2022) 17(1):80. doi: 10.1186/s13020-022-00636-1 35765020 PMC9237974

[19] Tang Y Liang Y Wang X Deng L. Analysis of acupoints combination for cancer-related anorexia based on association rule mining. Evid Based Complement Alternat Med (2022) 2022:4251458. doi: 10.1155/2022/4251458 36304134 PMC9596268

[20] Chen J Xie Y Lin Q Qian Z Feng J Zhang J. Investigating acupoint selection and combinations of acupuncture for tic disorders: an association rule mining and network analysis study. Front Neurol (2022) 13:894951. doi: 10.3389/fneur.2022.894951 35756940 PMC9226724

[21] World Health Organization. WHO regional office for the Western Pacific, WHO international standard terminologies on traditional medicine in the Western Pacific Region. Manila, Philippines: World Health Organization (2007).

[22] Yang JW Shao JK Wang Y Liu Q Liang JW Yan SY. Effect of acupuncture on postoperative ileus after laparoscopic elective colorectal surgery: A prospective, randomised, controlled trial. EClinicalMedicine (2022) 49:101472. doi: 10.1016/j.eclinm.2022.101472 35747183 PMC9156985

[23] Zhang Z Wang C Li Q Zhang M Zhao H Dong L. Electroacupuncture at ST36 accelerates the recovery of gastrointestinal motility after colorectal surgery: a randomised controlled trial. Acupuncture m Med (2014) 32(3):223–6. doi: 10.1136/acupmed-2013-010490 24739815

[24] Long Y Zhang Z Huang Z Chen J Xie X. Clinical observation of electroacupuncture at yuan-primary point and luo-connecting point of the large intestine meridian of hand yangming in the treatment of postoperative intestinal obstruction in colorectal cancer. J Guangzhou Univ Chin Med (2021) 38(03):518–23. doi: 10.13359/j.cnki.gzxbtcm.2021.03.016

[25] Zhu W Wang Y Gao C Zhou J Lu J Chen H. The effect of electroacupuncture treatment on postoperative recovery in patients with colon cancer. Shanghai J Acupuncture Moxibustion (2021) 40(04):416–20. doi: 10.134600/j.issn.1005-0957.2021.04.0416

[26] Liang Z Yang J Wang Z Zhang C. The effect of combined acupuncture with the concept of enhanced recovery after surgery on the recovery of gastrointestinal function after colorectal cancer surgery. Chin J Med Guide (2020) 17(25):125–128+ 140.

[27] Wang X Wang Z Lai Y. Clinical study on the treatment of postoperative gastrointestinal dysfunction in colorectal cancer with lao shi needles. Modem Distance Educ Traditional Chin Med (2022) 20(15):105–7.

[28] Zhang K Zhang Y Feng Y Zhao J Lu J. Clinical study on the effect of acupuncture therapy on the recovery of gastrointestinal function after colorectal cancer surgery. Health Reading (2022) 1:276–8.

[29] Li D Yang Z Qiu F Li G Shi Z Yang Z. Application effect of acupuncture therapy combined with enhanced recovery after surgery and nutritional support in the perioperative period of colorectal cancer. Guangxi Med J (2018) 40(08):872–5.

[30] Xie J. Influence of traditional chinese nursing on postoperative recovery and complications in patients with rectal cancer. New Traditional Chin Med (2014) 46(12):226–8.

[31] Tong W Ayi G Xu L. Effect of acupoint acupuncture on gastrointestinal motility after rectal cancer surgery. Chin J Med Guide (2014) 20(12):39–41. doi: 10.13862/j.cnki.cn43-l 446/r.2014.12.0l 4

[32] Zhang S Du Y. Effects of warm acupuncture on gastrointestinal and immune function in patients after colorectal cancer surgery. Chin Acupuncture (2011) 31(06):513–7. doi: 10.13703/j.0255-2930.2011.06.013 21739693

[33] Wang Y Li TH. Research on promoting gastrointestinal function recovery after rectal cancer surgery by electroacupuncture at distant and near points. Clin J acupuncture moxibustion Moxibustion. (2019) 35(04):26–8.

[34] Xue Y. Clinical study of bilateral zusanli electroacupuncture for rapid recovery after laparoscopic radical resection of rectal cancer. Guangzhou , China: Guangzhou University of Chinese Medicine (2020). doi: 10.27044/d.cnki.ggzzu.2020.000610

[35] Li Y. Clinical observation of electroacupuncture treatment for gastrointestinal function recovery after colorectal cancer surgery. Dalian, China: Dalian Medical University (2017). doi: 10.26994/d.cnki.gdlyu.2017.000249

[36] Sun H Zhang B Qian H Chen Z. Effects of warm acupuncture intervention on immune function and intestinal microbiota in patients after radical resection of colorectal cancer. Acupuncture Res (2021) 46(07):592–7. doi: 10.13702/j.1000-0607.200647 34369680

[37] Li W Zhao J Ran D. Effects of different frequency electroacupuncture on gastrointestinal function, stress response , cytokines, and immune mechanisms in patients undergoing total intravenous anesthesia laparoscopic radical resection of rectal cancer. Chin J Med Guide (2021) 27(07):93–96+99. doi: 10.13862/j.cnki.cn43-1446 /r.2021.07.018

[38] Yang J. Study on the effect of combined electroacupuncture at he points on gastrointestinal function in patients after colorectal cancer surgery. Guangzhou , China: Guangzhou University of Chinese Medicine (2011).

[39] Zhou Y. Clinical study of electroacupuncture promoting postoperative gastrointestinal function recovery after laparoscopic radical resection of rectal cancer. Jinan, China: Shandong University of Traditional Chinese Medicine (2021). doi: 10.27282/d.cnki.gsdzu.2021.000087

[40] Xiao C. Clinical study of electroacupuncture promoting postoperative gastrointestinal function recovery in patients with colorectal cancer. Changsha, China: Hunan University of Chinese Medicine (2014).

[41] Wang H. Influence of acupuncture on postoperative gastrointestinal function recovery in patients with colorectal cancer undergoing rapid rehabilitation surgery. Nanjing , China: Nanjing University of Chinese Medicine (2011).

[42] Xiao C. Study on the promotion of postoperative gastrointestinal function recovery and serum miR-19a by zusanli acupuncture in patients with colorectal cancer. Guangzhou , China: Guangzhou University of Chinese Medicine (2016).

[43] Si J Ding Y. Clinical study on electroacupuncture for the recovery of gastrointestinal function after radical surgery for colorectal cancer. J Pract Traditional Chin Med (2015) 31(08):754–5.

[44] Niu C Li D Gao Y. Effect of electroacupuncture at acupoints on intestinal peristalsis after radical surgery for colorectal cancer. J Changchun Univ Traditional Chin Med (2008) 01):83. doi: 10.13463/j.cnki.cczyy.2008.01.035

[45] Xiao L Zhou B Zhang J Chai Z Yun T Zhao G. Clinical study on improving postoperative intestinal function in elderly patients with colon cancer treated by tiaofei yichang acupuncture method. J Shandong Univ Traditional Chin Med (2016) 35(08):701–4. doi: 10.16295/j.cnki.0257-358x.2016.08.0ll

[46] Wang T Meng J Mai S. Effects of electroacupuncture at different time points on postoperative intestinal function in patients undergoing radical resection of colorectal cancer under combined intravenous anesthesia. Acupuncture Res (2018) 43(12):797–800. doi: 10.13702/j.1000-0607.170381 30585459

[47] Mai S Meng J Wang W Lang S. Effects of electroacupuncture preconditioning on intestinal function in patients undergoing surgery for colorectal cancer. Chin Acupuncture (2017) 37(05):483–7. doi: 10.13703/j.0255-2930.2017.05.008 29231608

[48] Meng Z Garcia MK Chiang JS Peng HT Shi YQ Fu J. Electro-acupuncture to prevent prolonged postoperative ileus: a randomized clinical trial. World J Gastroenterol (2010) 16(1):104–11. doi: 10.3748/wjg.vl6.il.104 PMC2799905 20039456

[49] Feng D. Effects of transcutaneous acupoint electrical stimulation preconditioning on postoperative gastrointestinal function recovery in patients undergoing laparoscopic colon cancer surgery. Hefei, China: Anhui Medical University (2020). doi: 10.27374/d.cnki.gwnyy.2020.000294

[50] Wang D. Effects of transcutaneous acupoint electrical stimulation (TEAS) on postoperative gastrointestinal function and inflammatory cytokines in patients undergoing laparoscopic surgery for sigmoid colon and rectal cancer. Chengdu, China: Chengdu University of Traditional Chinese Medicine (2021). doi: 10.26988/d.cnki.gcdzu.202l.000418

[51] Huang W Yu T Long W Xiao J. Application of transcutaneous acupoint electrical stimulation combined with transversus abdominis plane block in enhanced recovery after surgery. Acupuncture Res (2018) 43(10):611–615+621. doi: 10.13702/j.1000-0607.180005 30365254

[52] Cai W Yi S Hao J Dong B. Effects of transcutaneous acupoint electrical stimulation-assisted anesthesia on gastrointestinal hormones and immune function in patients undergoing laparoscopic rectal cancer surgery. Chin J Oncol Rehabil (2021) 28(01):37–40.

[53] Ren Z Liang W Yin N. Effects of transcutaneous acupoint electrical stimulation on postoperative gastrointestinal function in patients undergoing laparoscopic radical resection of rectal cancer. Jiangsu Med (2021) 47(10):1017–1019+1023. doi: 10.19460/j.cnki.0253-3685.2021.10.012

[54] Song Y Song W Ding H Huang Y. Effects of transcutaneous acupoint electrical stimulation on gastrointestinal hormones and immune function in patients undergoing laparoscopic radical resection of rectal cancer. Chin J Traditional Chin Med (2020) 35(01):210–4. doi: 10.16368/j.issn.1674-8999.2020.01.046

[55] Xu Y Cai B Shen P. Pulsed signal stimulation of acupoints promotes postoperative intestinal function recovery after dixon surgery. Shanghai J Traditional Chin Med (2006) 08):36–7. doi: 10.16305/j.1007-1334.2006.08.019

[56] Yue S. Effects of transcutaneous acupoint electrical stimulation on anesthesia and postoperative recovery in patients undergoing laparoscopic radical resection of colorectal cancer. Taiyuan, China: Shanxi Medical University (2021). doi: 10.27288/d.cnki.gsxyu.2021.000718

[57] Fu H Zhao Z Du W Wang J Jia D. Effects of transcutaneous acupoint electrical stimulation on pain and rapid recovery after laparoscopic radical resection of colorectal cancer. Chin J Modern Doctors (2022) 60(22):15–18+47.

[58] Zhang H Hao J Bai Y Lu Y. Effects of transcutaneous acupoint electrical stimulation on postoperative intestinal function in patients with colorectal tumors. Modern Oncol (2020) 28(04):611–4.

[59] Xu Y. Effects of general anesthesia combined with transcutaneous acupoint electrical stimulation on stress response and postoperative recovery in patients undergoing laparoscopic radical resection of rectal cancer. Infection Inflammation Repair (2021) 22(02):106–9.

[60] Wei Q Pang Y Zuo H Mo X Wu R Li X. Effects of transcutaneous acupoint electrical stimulation combined with chewing gum on gastrointestinal function in patients after colorectal cancer surgery. Chin J Modern Nurs (2019) 25(21):2746–9. doi: 10.3760/cma.j.issn.1674-2907.2019.21.024

[61] Liang Z Yang J Wang Z Zhang C. Effects of the combined concept of enhanced recovery after surgery and acupuncture on postoperative gastrointestinal function recovery in patients with colorectal cancer. China Med Herald (2020) 17(25):125–128+140.

[62] Meng ZQ Garcia MK Chiang JS Peng HT Shi YQ Fu J. Electro-acupuncture to prevent prolonged postoperative ileus: a randomized clinical trial. World J Gastroenterol (2010) 16(1):104–11. doi: 10.3748/wjg.v16.i1.104 PMC2799905 20039456

[63] Zhu SF Guo H Zhang RR Zhang Y Li J Zhao XL. Effect of electroacupuncture on the inflammatory response in patients with acute pancreatitis: an exploratory study. Acupunct Med (2015) 33(2):115–20. doi: 10.1136/acupmed-2014-010646 25520280

[64] Zhang Z Yu Q Zhang X Wang X Su Y He W. Electroacupuncture regulates inflammatory cytokines by activating the vagus nerve to enhance antitumor immunity in mice with breast tumors. Life Sci (2021) 272:119259. doi: 10.1016/j.lfs.2021.119259 33636172

[65] Huang W Long W Xiao J Zhao G Yu T. Effect of electrically stimulating acupoint, Zusanli (ST 36), on patient's recovery after laparoscopic colorectal cancer resection: a randomized controlled trial. J Tradit Chin Med (2019) 39(3):433–9. 32186016

[66] Yang NN Ye Y Tian ZX Ma SM Zheng Y Huang J. Effects of electroacupuncture on the intestinal motility and local inflammation are modulated by acupoint selection and stimulation frequency in postoperative ileus mice. Neurogastroenterol Motil. (2020) 32(5):e13808. doi: 10.1111/nmo.13808 32114712

[67] Liu M Zhang S Gai Y Xie M Qi Q. Changes in the interstitial cells of cajal and immunity in chronic psychological stress rats and therapeutic effects of acupuncture at the zusanli point (ST36). Evid Based Complement Alternat Med (2016) 2016:1935372. doi: 10.1155/2016/1935372 27594888 PMC4987473

[68] Liu S Wang ZF Su YS Ray RS Jing XH Wang YQ. Somatotopic organization and intensity dependence in driving distinct NPY-expressing sympathetic pathways by electroacupuncture. Neuron (2020) 108(3):436–450.e7. doi: 10.1016/j.neuron.2020.07.015 32791039 PMC7666081

[69] Gao ZX Wang W Lv E Zhao JR. Effect and mechanism of needling shangjuxu acupoint on alleviating chronic visceral hypersensitivity. J Shanxi Datong Univ (Natural Sci Edition). (2010) 26(03):42–4.

[70] Yang JJ. The effects of electroacupuncture at the lower He-sea points on gastrointestinal function in postoperative patients with colorectal cancer. Guangzhou, China: Guangzhou University of Chinese Medicine (2011).

[71] Wang QL Lin ZG Cai GH Chen YQ. Clinical observation of warm acupuncture at the lower He-sea points for the treatment of incomplete intestinal obstruction. Guangxi J Traditional Chin Med (2019) 42(06):38–40.

[72] Fei ZQ Chen DX Yao TQ Jing HB Wang YW Tian YX. Effect of acupressure stimulation of Zusanli, Shangjuxu, and Xiajuxu acupoints on gastrointestinal function recovery after laparoscopic cholecystectomy. Beijing J Traditional Chin Med (2022) 41(03):326–8.

[73] Ai K Wu JF Qi F Zhang H Yi XQ Ling X. Effect of electroacupuncture at "Zusanli" (ST 36) and other points on TNF-α and NF-κB levels in rats with acute gastric mucosal injury. World J Acupuncture-Moxibustion. (2016) 26(04):26–32. doi: 10.1016/S1003-5257(17)30019-3 29231528

[74] Zhang H Wu JF Qi F Yin XT Ai K Yi XQ. Comparative study of the effects of electroacupuncture at different acupoints on HMGB1 and nAchR α7 in a rat model of acute gastric mucosal injury. Chin Acupuncture Moxibustion. (2016) 36(10):1071–6. doi: 10.13703/j.0255-2930.2016.10.018 29231528

[75] Xiang ZY. Comparative study on the treatment of ulcerative colitis in rats with electroacupuncture at Shangjuxu and Quchi points: A preliminary exploration of the concept of "Curing Viscera Diseases by the lower He-sea point". Changsha, China: Hunan University of Traditional Chinese Medicine (2004).

[76] Liang C Wang KY Gong MR Li Q Yu Z Xu B. Electro-acupuncture at ST37 and ST25 induce different effects on colonic motility via the enteric nervous system by affecting excitatory and inhibitory neurons. Neurogastroenterol Motil. (2018) 30(7):e13318. doi: 10.1111/nmo.13318 29488287

[77] Noguchi E. Acupuncture regulates gut motility and secretion via nerve reflexes. Auton Neurosci (2010) 156(1-2):15–8. doi: 10.1016/j.autneu.2010.06.010 20663717

[78] Xing JJ Zeng BY Li J Zhuang Y Liang FR. Acupuncture point specificity. Int Rev Neurobiol (2013) 111:49–65. doi: 10.1016/B978-0-12-411545-3.00003-1 24215917

[79] Langevin HM Wayne PM. What is the point? The problem with acupuncture research that no one wants to talk about. J Altern Complement Med (2018) 24(3):200–7. doi: 10.1089/acm.2017.0366 PMC6421999 29493256

